# Assessing potential sources of clustering in individually randomised trials

**DOI:** 10.1186/1471-2288-13-58

**Published:** 2013-04-16

**Authors:** Brennan C Kahan, Tim P Morris

**Affiliations:** 1 MRC Clinical Trials Unit, Aviation House, 125 Kingsway, London, WC2B 6NH, UK

**Keywords:** Clustering, Randomised controlled trials, Unadjusted analyses, Therapeutic trials, Surgical trials, Multicenter trials

## Abstract

**Background:**

Recent reviews have shown that while clustering is extremely common in individually randomised trials (for example, clustering within centre, therapist, or surgeon), it is rarely accounted for in the trial analysis. Our aim is to develop a general framework for assessing whether potential sources of clustering must be accounted for in the trial analysis to obtain valid type I error rates (non-ignorable clustering), with a particular focus on individually randomised trials.

**Methods:**

A general framework for assessing clustering is developed based on theoretical results and a case study of a recently published trial is used to illustrate the concepts. A simulation study is used to explore the impact of not accounting for non-ignorable clustering in practice.

**Results:**

Clustering is non-ignorable when there is both correlation between patient outcomes within clusters, and correlation between treatment assignments within clusters. This occurs when the intraclass correlation coefficient is non-zero, and when the cluster has been used in the randomisation process (e.g. stratified blocks within centre) or when patients are assigned to clusters after randomisation with different probabilities (e.g. a surgery trial in which surgeons treat patients in only one arm). A case study of an individually randomised trial found multiple sources of clustering, including centre of recruitment, attending surgeon, and site of rehabilitation class. Simulations show that failure to account for non-ignorable clustering in trial analyses can lead to type I error rates over 20% in certain cases; conversely, adjusting for the clustering in the trial analysis gave correct type I error rates.

**Conclusions:**

Clustering is common in individually randomised trials. Trialists should assess potential sources of clustering during the planning stages of a trial, and account for any sources of non-ignorable clustering in the trial analysis.

## Background

Many randomised controlled trials (RCTs) involve some form of clustering. Common examples include cluster randomised trials (where clusters themselves are randomised to treatment arms), multicentre trials (where patients are clustered within centres), and therapist or surgical trials (where patients are clustered within surgeons or therapists). It is well known that while ignoring clustering in the analysis of a RCT will give asymptotically unbiased estimates of treatment effect [1], it can lead to incorrect estimates of the standard error (SE), and therefore incorrect type I error rates. Perhaps the most well-known example of this is cluster randomised trials, where failure to appropriately adjust for the clusters in the analysis will result in SEs that are too small, leading to type I error rates that are too large [2,3].

Clustering is also common in individually randomised trials (e.g. multicentre or therapist/surgical trials). Lee and Thompson found that 90% of the individually randomised trials they reviewed involved some form of clustering [4]. Despite the frequency of clustering in randomised trials, recent articles have highlighted the lack of awareness many trialists have regarding the issues clustering presents for the analysis [4-6]; for example, Lee and Thompson found only 4/38 trials (11%) adjusted for clustering (and 3 of the 4 trials that did adjust for clustering did not account for all sources of clustering). This may lead to incorrect conclusions regarding treatment efficacy, and could potentially lead to ineffective treatments being adopted, or effective treatments being discarded. Despite this, there are some situations where not accounting for clustering in the analysis will still lead to valid results (for example, as long as the randomisation is not stratified on centre in a multicentre trial, an unadjusted analysis will still give correct type I error rates [7]).

It is important to clarify under what conditions clustering must be accounted for in the analysis of a RCT so that trialists are able to perform an appropriate analysis. The aim of this paper is to develop a framework to assess when non-ignorable clustering occurs in individually randomised trials (i.e. clustering that must be accounted for in the analysis in order to obtain valid type I error rates), and examine its potential impact on trial results.

## Methods

### Definition of clustering

In the context of a RCT, we define clustering as when observations are grouped together based upon common attributes. This includes standard examples of clustering such as multicentre trials (where patients are grouped together within centres), crossover trials (where observations are grouped within patients), and trials where the intervention is a type of surgery or therapy and patients are grouped together by surgeon or therapist.

Our definition of clustering also includes some non-standard situations (for example when patients are grouped according to baseline factors, for example age and sex, and then randomised within these strata). Although this type of scenario would not generally be regarded as clustering in the typical sense, it affects the analysis in exactly the same way as standard clustering, and therefore we have included it in our definition for completeness.

Clustering can also be defined as either pre- or post-randomisation, and determining whether clustering is non-ignorable will depend on when it occurs. Pre-randomisation clustering occurs when patients are grouped into clusters and then randomised, for example when patients present to different centres and are randomised upon presentation. Post-randomisation clustering occurs when patients are randomised and then assigned to clusters, for example when they are randomised to a type of surgery and then assigned to a specific surgeon.

It should be noted that whether clustering is considered to be pre- or post-randomisation largely depends on the timing of the randomisation. For example, if patients are randomised and then are assigned to a therapist, therapist would be considered post-randomisation clustering. However, if therapist is used as a stratification factor in the randomisation, and patients are assigned to a therapist and then randomised, therapist would be considered pre-randomisation clustering.

### Theoretical definition of non-ignorable clustering

Based on results from Parzen et al. [7], it can be shown under what circumstances clustering will be non-ignorable. In the presence of clustering, the true variance of the treatment effect for a continuous outcome can be written as:

$$\mathrm{Var}\left(\hat{\beta }\right)=V_{0}+V_{E}$$

where $\hat{\beta }$ is the treatment effect, *V*_*0*_ is the usual (asymptotic) variance of the treatment effect when clustering is not present, and *V*_*E*_ is an additional factor based on the clustering, which can be either positive or negative. For further mathematical details of this expression, please see Parzen et al. [7] (it should be noted that our notation differs slightly to theirs).

When *V*_*E*_=0, the estimate of variance ignoring clustering will be unbiased, and clustering will be ignorable. However, when *V*_*E*_≠0 the estimate of variance ignoring clustering will be either biased upwards (if *V*_*E*_<0) or downwards (if *V*_*E*_>0). When *V*_*E*_≠0 the clustering is therefore non-ignorable, and needs to be accounted for in the analysis in order to obtain valid results.

*V*_*E*_ is a function of the correlation between outcomes for patients in the same cluster (generally referred to as the intraclass correlation coefficient or ICC), and the correlation between treatment assignments for patients in the same cluster. If either of these correlations are 0, *V*_*E*_ will also be 0 and the clustering will not need to be included in the analysis in order to obtain valid type I error rates (although it still may be preferable to adjust for this type of clustering as it could increase power or precision). However, if both of these correlations are non-zero then *V*_*E*_≠0, and the clustering will be non-ignorable, and must be accounted for in the analysis. Parzen et al. showed similar results for binary and time-to-event outcomes [7].

Assuming that the ICC is positive, then *V*_*E*_>0 when the correlation between treatment assignments is positive (leading to the SE for treatment being biased downwards), and *V*_*E*_<0 when the correlation between treatment assignments is negative (leading to the SE being biased upwards).

### Non-ignorable clustering in practice

Correlation between patient outcomes within a cluster may occur for two primary reasons. The first is that patients with similar characteristics may be more likely to present to the same cluster (e.g. patients with similar socio-economic status may be share the same hospital). The second possibility is that the clusters themselves exert some influence on outcome (e.g. patients within a certain hospital may be more likely to have a positive outcome due to different processes of care or quality of hospital staff).

Treatment assignments between patients in the same cluster will be correlated if patients in certain clusters are more likely to be in a certain treatment group [7]. A simple example of this is a cluster randomised trial, in which all patients in a cluster receive the same treatment. If we know the treatment group of one patient, we then know the treatment group of all patients in that cluster, leading to a correlation between treatment assignments of 1. Conversely, in a 2×2 crossover trial where each patient receives one treatment in the first period and the other treatment in the second period, if we know which of the two treatments they received in the first period than we also know which treatment they received in the second period, indicating the correlation between treatment assignments is -1. Stratified permuted blocks within clusters leads to negative correlation between treatment assignments (for each patient assigned to a specific treatment, it makes it less likely that future patients will be assigned to that same treatment). The exact correlation for stratified permuted blocks is $\frac{-1}{n-1}$, where *n* is the block size. This indicates that the correlation will always be between -1 and 0. Simple randomisation (where all patients are randomised independently) leads to a correlation of 0.

As discussed in the previous section, non-ignorable clustering occurs when *both* the ICC *and* the correlation between treatment assignments within a cluster is non-zero. However, if *either* the ICC *or* the correlation between treatment assignments within a cluster is 0, the clustering is ignorable, and valid SEs and type I error rates can be obtained regardless of whether the clustering is accounted for in the analysis (although not adjusting for clustering may lead to a loss of power).

### Determining whether clustering is non-ignorable

It is important to identify any sources of non-ignorable clustering during the planning stages of the trial to ensure they can be adequately adjusted for in the analysis. In order to determine whether clustering is non-ignorable, we first need to determine whether the ICC and the correlation between treatment assignments is non-zero.

The ICC will not generally be known prior to the trial commencing (unless previous data is available). It is possible to estimate the ICC based on the trial data, however this type of data-dependent model selection has been shown to give poor results, and could potentially inflate the type I error [8]. Therefore, in order to avoid erroneously excluding non-ignorable clustering from the analysis, we suggest that the ICC should be assumed to be non-zero unless there is evidence to the contrary or strong reasons for suspecting it is 0.

Determining whether the correlation between patient assignments within clusters is non-zero depends on whether the clustering is pre or post-randomisation. If it is pre-randomisation clustering (i.e. patients are grouped into clusters and then randomised) then this correlation will be non-zero if the clustering is used in the randomisation process. Examples of clusters being used in the randomisation process include cluster randomised trials (where the clusters themselves are randomised), randomisation that balances on patient factors (such as recruiting centre or baseline prognostic factors), or any trials where patient outcomes are measured and analysed at several time points (as happens in crossover trials and longitudinal studies). If the cluster is not used in the randomisation process then the clustering is ignorable, and type I error rates will be correct regardless of whether the cluster is accounted for in the analysis. For example, an analysis of a multicentre trial ignoring centre effects will give unbiased SEs and correct type I error rates, provided that centre was not balanced on in the randomisation.

For post-randomisation clustering, the correlation between treatment assignments will be 0 if patients in both treatment groups have an equal chance of being assigned to the same clusters (for example, if ward nurses are responsible for the care of patients, but have an equal chance of treating patients from each treatment arm). If the treatment groups are assigned to clusters with different probabilities, then the correlation will be non-zero. Examples of this include therapy or surgery trials when therapists and surgeons only treat patients in one arm. If therapists or surgeons treat patients in both arms, but are more likely to treat patients from a specific arm, this will still result in correlation between treatment assignments within clusters.

### Case study – the FASTER trial

We use the FASTER trial (Function After Spinal Treatment: Exercise and Rehabilitation) [9] as a case study for assessing sources of clustering in a real RCT. FASTER was a 2×2 factorial trial designed to assess the impact of a series of rehabilitation classes or an educational booklet on outcomes following back surgery. Although two treatments were assessed in this trial, we focus here on rehabilitation. The primary outcome was the Oswestry disability index, which assesses how much impact a patient’s back pain has on their functional ability. Table 1 shows the structure of the clustering for FASTER.

**Table 1:** Example of the potential clustering structure in FASTER

**Patient**	**Centre**	**Procedure**	**Surgeon**	**Rehab**	**Class attended**
1	1	Dis	1	Y	A
2	1	Dis	1	N	-
3	1	Dis	2	Y	A
4	1	Dis	2	N	-
5	1	Dec	1	Y	B
6	1	Dec	1	N	-
8	1	Dec	2	Y	C
9	1	Dec	2	N	-
10	2	Dis	1	Y	A
11	2	Dis	1	N	-
12	2	Dec	3	Y	C
13	2	Dec	3	N	-

Patients are randomised to either receive rehab or not. Patients are clustered within centre, and are stratified by the type of surgical procedure they receive. Surgeons operate across centres. Patients are able to choose which rehab class they attend.

338 patients were recruited from seven centres, and operations were performed by one of 23 surgeons involved in the trial. The majority of surgeons performed operations across multiple centres. Randomisation was stratified by operating surgeon and the type of surgery (discectomy or decompression), but not by recruiting centre.

Six weeks after randomisation, patients assigned to the rehabilitation group were expected to attend rehabilitation classes for six weeks, with two classes per week. Patients generally attended classes at the centre where the surgery was performed, but could choose to attend at another centre if they wished.

During the planning stages of the trial, the following sources of clustering would need to be assessed to determine whether they are non-ignorable:

#### Recruiting centre (pre-randomisation clustering)

Patients from the same centre may have similar outcomes (implying a positive ICC value). However, because the recruiting centre was not used in the randomisation process, the correlation between treatment assignments within centres will be 0, indicating that this is not a form of non-ignorable clustering. However, it should be noted that although it is not strictly necessary to adjust for centre-effects in this situation, if the ICC is high then adjustment for centre will increase power.

#### Surgeon (pre-randomisation clustering)

Patient outcomes may vary based on their surgeon, as more skilled surgeons may produce better outcomes or be required to treat the most difficult cases, resulting in a non-zero ICC value. Because surgeon was a stratification factor, the correlation between patient assignments within surgeon will also be non-zero, indicating that surgeon is a source of non-ignorable clustering. Ignoring surgeon in the analysis could lead to a type I error rate that was too conservative.

#### Type of surgery - discectomy or decompression (pre-randomisation clustering)

Type of surgery may affect back pain, resulting in a positive ICC value. This variable was balanced on in the randomisation process, creating correlation between patient assignments within each level of surgery type, meaning it is a source of non-ignorable clustering. Ignoring type of surgery in the analysis may lead to a type I error rate that was too conservative.

#### Rehabilitation classes (post-randomisation clustering)

Because patients in only one treatment group attended rehabilitation classes, the correlation between treatment assignments within classes will be positive. It is possible that the quality of classes may vary, and patients in some classes have better outcomes than patients in other classes. This is therefore a form of non-ignorable clustering. Ignoring rehabilitation class in the analysis could lead to inflation of the type I error rate.

Of the four sources of clustering, we have identified three as non-ignorable. Two of the non-ignorable sources of clustering were pre-randomisation, and one was post-randomisation. Because some sources of clustering would lead to upward bias in the SE, and others to downward bias, it is possible the different sources of clustering may cancel each other out to some degree. However it is very unlikely that the bias in the SE will be cancelled out entirely, and so ignoring all sources of clustering in the analysis will likely lead to bias of unknown magnitude and direction. Therefore, we recommend all sources of non-ignorable clustering be accounted for. The analysis model for FASTER would need to account for surgeon, type of surgery, and which rehabilitation class they attended. It would be important that this assessment be done during the planning stages of the trial, so that the relevant information could be collected during the trial (e.g. which rehabilitation class the patient attended).

The FASTER trial received ethical approval from Hammersmith and Queen Charlotte’s and Chealsea Hospitals research Ethics Committee, and was carried out in compliance with the Helsinki Declaration.

### Simulation study

We performed a simulation study to assess the impact of clustering on study results. Simulations imitated a trial of therapeutic intervention, where patients were randomised, and then assigned to a therapist (post-randomisation clustering).

Data were generated from the following model (which can be used to describe both pre- and post-randomisation clustering):

$$y_{\mathrm{ij}}=\alpha +\beta_{\mathrm{treat}}x_{\mathrm{ij}}+u_{j}+e_{\mathrm{ij}}$$

where *y*_*ij*_ is a continuous outcome for patient *i* from therapist *j*, *α* is an intercept, *β*_*treat*_ is the treatment effect and *x*_*ij*_ a binary variable indicating which treatment arm the patient was in, *u*_*j*_ the therapist effect for therapist *j*, and *e*_*ij*_ a random error term for patient *i* from therapist *j*. We generated *e*_*ij*_ from a normal distribution with mean 0 and variance 1, and *u*_*j*_ from a normal distribution with mean 0, and *σ*^*2*^ (where *σ*^*2*^ was set to give the desired ICC). We generated *e*_*ij*_ and *u*_*j*_ independently. *β*_*treat*_ was set to 0 for all simulations. It should be noted that the choice of variance for *e*_*ij*_ is arbitrary and has no effect on results.

We varied the following parameters:

•10 and 50 therapists were used

•two different sample sizes were used for each number of therapists. For 10 therapists we used sample sizes of 100 and 200, and for 50 therapists we used 500 and 1000 patients (an average of 10 and 20 patients per therapist respectively for both scenarios)

•ICC values of 0, 0.05, and 0.10 were used;

•Patients were assigned to different therapists after randomisation in one of three ways:

○ Therapists treated patients in both treatment arms equally. Patients were assigned to therapists with equal probability.

○ Therapists treated patients in both treatment arms, but were more likely to see patients from a certain treatment arm. Therapists were split into two groups, with an equal number of therapists in each. Patients in the first treatment arm were randomly assigned to a therapist from the first set with a probability of 80%, and to a therapist in the other set with a probability of 20%. Patients in the second treatment arm were assigned to the therapists with the reverse probabilities.

○ Therapists only treated patients in one treatment arm (with an equal number of therapists per arm). Patients in each treatment arm were randomly assigned to one of the therapists treating patients in their treatment arm only with equal probability.

We used 5000 replications for each of the 36 simulated scenarios. For each scenario we performed two analyses; the first adjusted for therapist effects, whereas the second did not. For each analysis method we assessed the type I error rate. Unadjusted analyses were performed using a linear regression model with the treatment assignment as the only covariate. When therapists treated patients in both arms (regardless of whether they treated both arms equally), adjusted analyses were performed using a linear regression model with treatment as a covariate, and therapist included as a fixed effect, using indicator variables.

When therapists treated only patients in one treatment arm, adjusted analyses were performed using cluster level summaries [2]; briefly, this involves calculating the mean outcome for each therapist, and fitting a linear regression model with these summaries as the outcome, and which treatment arm the therapist saw as a covariate. We used cluster-level summaries rather than a mixed-effects model in this scenario as there is evidence that mixed-effects models may not perform well in scenarios when clustering occurs within treatment arms, and there is a small number of clusters [2].

## Results

### Simulation results

**Figure 1:**
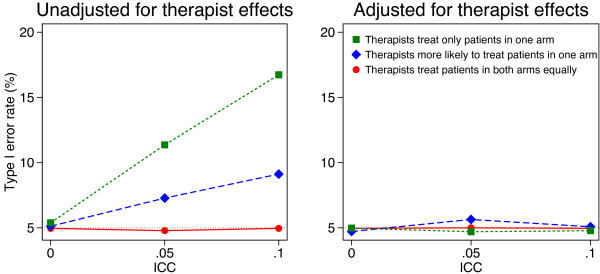
**Simulation results (10 therapists, 100 patients).** Continuous outcomes were generated based on a treatment effect of 0 and therapist effects and a random error term, both of which followed a normal distribution. Patients were assigned to therapist post-randomisation. An equal number of patients were assigned to each therapist, and we used 5000 replications for each scenario.

**Figure 2:**
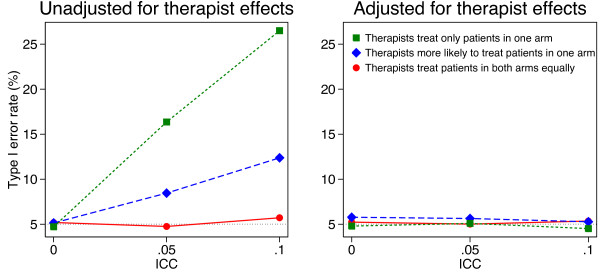
**Simulation results (10 therapists, 200 patients).** Continuous outcomes were generated based on a treatment effect of 0 and therapist effects and a random error term, both of which followed a normal distribution. Patients were assigned to therapist post-randomisation. An equal number of patients were assigned to each therapist, and we used 5000 replications for each scenario.

**Figure 3:**
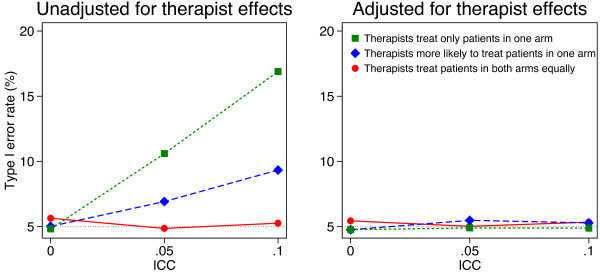
**Simulation results (50 therapists, 500 patients).** Continuous outcomes were generated based on a treatment effect of 0 and therapist effects and a random error term, both of which followed a normal distribution. Patients were assigned to therapist post-randomisation. An equal number of patients were assigned to each therapist, and we used 5000 replications for each scenario.

**Figure 4:**
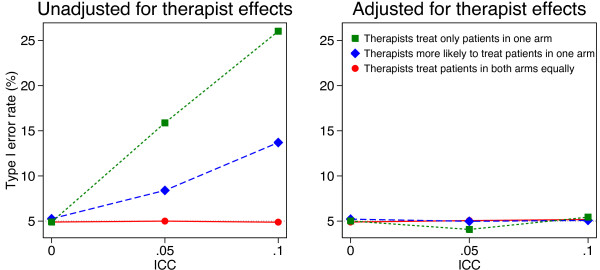
**Simulation results (50 therapists, 1000 patients).** Continuous outcomes were generated based on a treatment effect of 0 and therapist effects and a random error term, both of which followed a normal distribution. Patients were assigned to therapist post-randomisation. An equal number of patients were assigned to each therapist, and we used 5000 replications for each scenario.

Simulation results are shown in Figures 1, 2, 3 and 4. Results were similar across all cluster and sample size combinations. When clustering was ignorable (that is, when either the ICC was 0, or therapists treated patients from both arms equally), type I error rates were correct regardless of whether an adjusted or unadjusted analysis was used (mean 5.1 and 5.0% for unadjusted and adjusted analyses respectively).

When clustering was non-ignorable (ICC > 0 and patients were assigned to therapists with different probabilities depending on the treatment group they were in), unadjusted analyses led to inflated type I error rates. This was most pronounced when therapists only treated patients in one arm (mean 13.6 and 21.5% for ICC values of 0.05 and 0.10 respectively). Even when therapists treated patients in both arms, but were more likely to treat patients in one arm, type I error rates were inflated (mean 7.8 and 11.1% for ICC values of 0.05 and 0.10 respectively).

Conversely, adjusted analyses gave correct type I error rates in all scenarios with non-ignorable clustering (mean 5.1%, range across 24 scenarios 4.1 to 5.6%).

## Discussion

Clustering is common in randomised trials, and although it is well known in certain situations that clustering needs to be accounted for (e.g. cluster randomised and crossover trials), there is evidence that other types of clustering are not properly handled [4].

It is important for trialists to recognise when non-ignorable clustering occurs in order to appropriately adjust their analysis; failure to do so will lead to biased SEs and incorrect type I error rates. This is of particular concern for post-randomisation clustering, where the clustering may not always be immediately apparent. Our simulations show type I error rates could be inflated to over 20% if clustering is not accounted for in the analysis. The amount the type I error rate will be inflated partly depends on the ICC. Cooke et al. presented 45 ICCs from surgical trials and found that 42% were >0.05 and 16% were >0.20, which could lead to substantial inflations in the type I error rate [10]. It should be noted that even in the presence of a true treatment effect, not accounted for non-ignorable clustering in the analysis can overstate the evidence by giving confidence intervals that are too narrow.

One frequent situation of non-ignorable clustering that is not commonly recognised in practice is when trials balance randomisation within centres or by prognostic factors [11-13]. It has been shown that when the chosen balancing factors are associated with outcome, unadjusted analyses can lead to a large over-inflation of the SE, leading to type I error rates that are too low and a substantial reduction in power (>20% in some cases) [11].

A number of analysis methods are available that account for clustering. Common methods include fixed-effects, random-effects (or mixed-effects models), and generalised estimating equations, although a number of other methods exist that may be more appropriate in certain situations. The best method of adjustment will depend on the particular trial and the nature of the clustering. It is also possible to account for clustering using methods such as permutation tests [14,15], although this may be difficult in some scenarios (e.g. when clustering within treatment arm is present), and further research in this area is needed.

It is also important for trialists to recognise when clustering is ignorable, as there are some situations where an analysis that does not account for clustering may be preferable. For example, in a multicentre trial with very few patients per centre, adjusting for centre effects can be difficult and may lead to poor results. Therefore, if centre has not been balanced on in the randomisation scheme, the best analysis may be one that ignores centre. Another example is when there are multiple layers of clustering (e.g. patients within therapists within hospitals within countries); attempting to control for all levels of clustering can lead to an overly complex analysis that may not work well in practice. A simpler analysis adjusting for only the sources of non-ignorable clustering may be preferable.

**Figure 5:**
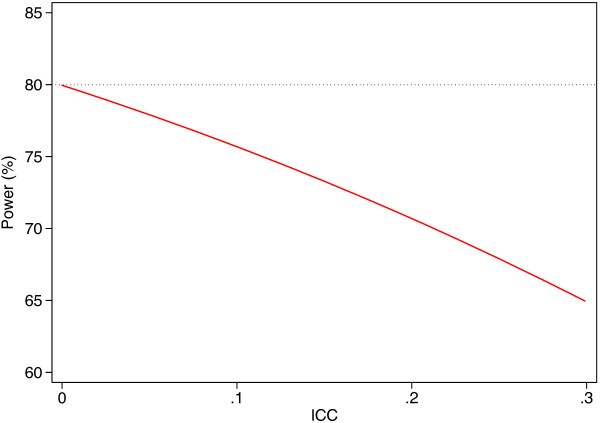
Loss in power from not accounting for ignorable clustering in the analysis.

When clustering is ignorable, unadjusted analysis will still give valid type I error rates. However, if the ICC is high, then an unadjusted analysis will lead to a loss of power, making it more difficult to detect a treatment effect. For example, in a therapy trial, if therapists have a large effect on the outcome, but are not any more likely to treat patients from a specific treatment group, then therapists are ignorable, and an analysis that does not account for therapist effects will still give valid results. However, adjusting for therapist in the analysis may be preferable as it will increase power. This is demonstrated in Figure 5, which shows the expected loss in power for trials which do not account for ignorable clustering in the trial (given the trial is powered at 80%). For large ICCs, the loss in power can be substantial; for example, ICCs of 0.10, 0.20, and 0.30 would lead to a reduction in power of 4%, 9%, and 15% respectively. Further work on the benefits of adjusting for factors associated with the outcome can be found elsewhere [16,17].

We have not considered the issue of treatment-by-cluster interactions (such as treatment-by-centre, or treatment-by-therapist). Although this could potentially be of interest in some scenarios, we agree with the ICH E9 guidelines, which suggest that treatment-by-centre interactions should not be involved in the primary analysis, but should rather be regarded as secondary analyses [18]. We have therefore been focused on issues pertaining to the primary analysis, and ignored treatment-by-cluster interactions. However, it should be noted that when there is clustering in one arm only, an analysis that accounts for the cluster is implicitly assuming a treatment-by-cluster interaction. For example, consider a trial of surgery vs. medical therapy, where patients are clustered within surgeons in one arm only. Under the null hypothesis of no treatment effect, assuming that outcomes vary by therapist implicitly assumes a treatment-by-surgeon interaction, meaning that surgery is beneficial compared to medical therapy for some surgeons and harmful for others.

It is generally recommended that adjustment for post-randomisation variables should be avoided in the analysis of RCTs in case they are on the causal pathway. For example, if a new treatment reduces mortality by lowering the patient’s blood pressure, adjustment for blood pressure at 6 months will lead to a biased result for mortality. In order to avoid adjustment for a variable on the causal pathway, trialists should adjust only for post-randomisation clustering when (1) it is part of the treatment (e.g. therapists or surgeons in a therapy or surgery trial); (2) patients are not assigned to clusters based on a post-randomisation factor, such as response to treatment, or an outcome at 6 weeks post-randomisation; and (3) clusters are not assigned to treatments based on the expected cluster-effect (e.g. therapists or surgeons are not assigned to a particular treatment group based on their skill level).

## Conclusion

Non-ignorable clustering is common in individually randomised trials, and can lead to large inflations in the type I error rate if not accounted for in the analysis. When planning a randomised trial it is important to give careful consideration to potential clustering, and to assess whether it is non-ignorable. This is important to do during the planning phases so that the appropriate data can be collected during the trial. Any sources of non-ignorable clustering should then be accounted for in the trial analysis in order to obtain correct confidence intervals and type I error rates.

## Abbreviations

FASTER: Function after spinal treatment: exercise and rehabilitation; ICC: Intracluster correlation coefficient; RCT: Randomised controlled trial; SE: Standard error

## Competing interests

The authors declare that they have no competing interests.

## Authors’ contributions

BK devised the study, performed the simulations, and wrote the first draft of the manuscript. TM input into the manuscript. Both authors had final approval for the decision to submit the manuscript for publication.

## Pre-publication history

The pre-publication history for this paper can be accessed here:

http://www.biomedcentral.com/1471-2288/13/58/prepub
